# Correction: Robust superhydrophobic and self-lubricating PTES-TiO_2_@UHMWPE fabric and its tribological properties

**DOI:** 10.1039/d3ra90079g

**Published:** 2023-08-22

**Authors:** Deke Li, Zhiguang Guo

**Affiliations:** a State Key Laboratory of Solid Lubrication, Lanzhou Institute of Chemical Physics, Chinese Academy of Sciences Lanzhou 730000 People's Republic of China zguo@licp.cas.cn +86-931-8277088 +86-931-4968105; b Hubei Collaborative Innovation Centre for Advanced Organic Chemical Materials, Ministry of Education Key Laboratory for the Green Preparation and Application of Functional Materials, Hubei University Wuhan 430062 People's Republic of China; c University of Chinese Academy of Sciences Beijing 100049 People's Republic of China

## Abstract

Correction for ‘Robust superhydrophobic and self-lubricating PTES-TiO_2_@UHMWPE fabric and its tribological properties’ by Deke Li *et al.*, *RSC Adv.*, 2017, **7**, 9169–9175, https://doi.org/10.1039/C6RA28255E.

The authors regret that an incorrect version of Fig. 1 was included in the original article. The correct version of Fig. 1 is presented below.

**Fig. 1:**
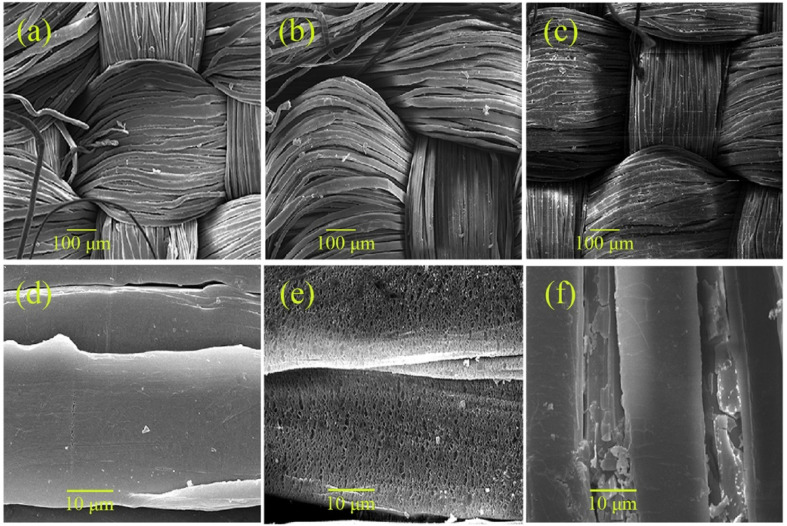
SEM images of pristine UHMWPE fabric (a), plasma-UHMWPE fabric (b) and PTES-TiO_2_@UHMWPE fabric (c), (d)–(f) are magnified images of (a)–(c), respectively.

An independent expert has viewed the corrected image and has concluded that it is consistent with the discussions and conclusions presented.

The Royal Society of Chemistry apologises for these errors and any consequent inconvenience to authors and readers.

## Supplementary Material

